# Accelerometer measured levels of moderate-to-vigorous intensity physical activity and sedentary time in children and adolescents with chronic disease: A systematic review and meta-analysis

**DOI:** 10.1371/journal.pone.0179429

**Published:** 2017-06-22

**Authors:** Rabha Elmesmari, John J. Reilly, Anne Martin, James Y. Paton

**Affiliations:** 1 School of Medicine, College of Medical, Veterinary, and Life Sciences, University of Glasgow, Glasgow, Scotland; 2 Al-Fatah Hospital, Medical School, Benghazi University, Benghazi, Libya; 3 University of Strathclyde, Physical Activity for Health Group, Glasgow, Scotland; 4 Usher Institute for Population Health Sciences and Informatics, Edinburgh, Scotland; University of Bremen, GERMANY

## Abstract

**Context:**

Moderate-to-vigorous physical activity (MVPA) and sedentary time (ST) are important for child and adolescent health.

**Objective:**

To examine habitual levels of accelerometer measured MVPA and ST in children and adolescents with chronic disease, and how these levels compare with healthy peers.

**Methods:**

Data sources: An extensive search was carried out in Medline, Cochrane library, EMBASE, SPORTDiscus and CINAHL from 2000–2017.

Study selection: Studies with accelerometer-measured MVPA and/or ST (at least 3 days and 6 hours/day to provide estimates of habitual levels) in children 0–19 years of age with chronic diseases but without co-morbidities that would present major impediments to physical activity. In all cases patients were studied while well and clinically stable.

**Results:**

Out of 1592 records, 25 studies were eligible, in four chronic disease categories: cardiovascular disease (7 studies), respiratory disease (7 studies), diabetes (8 studies), and malignancy (3 studies). Patient MVPA was generally below the recommended 60 min/day and ST generally high regardless of the disease condition. Comparison with healthy controls suggested no marked differences in MVPA between controls and patients with cardiovascular disease (1 study, n = 42) and type 1 diabetes (5 studies, n = 400; SMD -0.70, 95% CI -1.89 to 0.48, p = 0.25). In patients with respiratory disease, MVPA was lower in patients than controls (4 studies, n = 470; SMD -0.39, 95% CI -0.80, 0.02, p = 0.06). Meta-analysis indicated significantly lower MVPA in patients with malignancies than in the controls (2 studies, n = 90; SMD -2.2, 95% CI -4.08 to -0.26, p = 0.03). Time spent sedentary was significantly higher in patients in 4/10 studies compared with healthy control groups, significantly lower in 1 study, while 5 studies showed no significant group difference.

**Conclusions:**

MVPA in children/adolescents with chronic disease appear to be well below guideline recommendations, although comparable with activity levels of their healthy peers except for children with malignancies. Tailored and disease appropriate intervention strategies may be needed to increase MVPA and reduce ST in children and adolescents with chronic disease.

## Introduction

Children and adolescents with low levels of physical activity (PA) are at increased risk of becoming inactive adults, and of diseases which result from inadequate activity [1, 2]. In addition to low PA, there is increasing concern that high levels of sedentary time (ST) may also be common, and both low PA and high ST are important risk factors for chronic disease [3, 4]. Multiple national and international bodies have recommended a minimum of 60 minutes of moderate-to-vigorous intensity physical activity (MVPA) every day for school-age children and adolescents [5, 6]. These recommendations for the general population are usually also considered to be applicable to children and adolescents with chronic disease [7–9], with an understanding that usual levels of MVPA might be lower in such sub-groups, and achievement of the MVPA recommendation would be a slower and more gradual process than in the healthy population [7, 10–12].

Objective techniques such as accelerometry currently represent the most accurate methods for measuring the amount and intensity of PA and amount of ST [13, 14]. While there have been many studies on the levels and adequacy of MVPA and ST in healthy children and adolescents [15–18], there are surprisingly few such studies in children and adolescents with chronic disease. In fact, numerous previous studies and national PA surveillance programs have actually excluded children and adolescents with chronic disease.

The primary aim of the present study was therefore to examine whether children and adolescents with chronic disease met the current MVPA recommendation [1, 6, 19]. Secondary aims were to examine the amount of accelerometer-measured ST in children and adolescents with chronic diseases, and to determine whether accelerometer measured MVPA and ST in children and adolescents with chronic disease were different from those in healthy control or comparison groups. This systematic review provides evidence on whether levels of MVPA are adequate and ST excessive in children and adolescents with chronic disease.

## Methods

### Review governance and registration

A systematic review of the literature was performed in accordance with the Preferred Reporting Items for Systematic Reviews and Meta-Analyses (PRISMA) guidelines [20] as shown in S1 File. The review protocol was registered on PROSPERO (registration number CRD42015016783), the international prospective register for systematic reviews (http://www.crd.york.ac.uk/NIHR_PROSPERO) see S2 File.

### Study eligibility

**Inclusion criteria:** To be eligible for inclusion in the review, papers had to meet all of the following criteria as per PICOS principles: **P**opulation (children and adolescents aged from 0–19 years); **I**ntervention or exposure: chronic childhood disease (chronic disease defined as any physical health problem that lasts three months or more). The chronic diseases included were decided on following a scoping review and were cardiovascular disease, respiratory disease, diabetes type 1 or type 2, and malignancies); **C**omparison (where applicable): healthy children matched for relevant criteria (in particular age, gender); **O**utcome (accelerometer measured MVPA and/or ST of at least 3 days and 6 hours/day). All **s**tudy designs were considered eligible (cross-sectional, longitudinal, case-control studies and intervention studies if pre-intervention data could be extracted). We looked for original research studies, published in English, in peer-reviewed journals; a detailed description of the study eligibility criteria is given in S1 Table.

**Exclusion criteria:** Studies that included participants with co-morbid acute or chronic medical diseases or conditions that may have impacted their physical activity were excluded. The present study aimed to examine the subtle impact of chronic disease on MVPA and ST, not the more obvious impacts from co-morbidities that preclude physical activity (e.g. arising from injury or acute illness requiring bed rest, and chronic physical limitations from e.g. cerebral palsy). Because of the on going debate about whether obesity is a disease, studies in children with obesity were also excluded and are the subject of a separate report.

Since the aim of the review was to examine habitual levels of MVPA and ST, studies that measured these variables for less than 6 hours per day or over two days or less were excluded. Recommendations currently exist for habitual (overall) MVPA rather than MVPA during specific domains (e.g. the after school period) and so studies that focused only on specific periods of the day (e.g. school activity only, or outdoor activity only, or weekend or weekday activity only, or after-school only) were also excluded.

#### Search strategy

The literature search was conducted using the five most relevant electronic databases: MEDLINE OVID; Cochrane library; EMBASE; SPORTSDiscus and CINAHL. We searched from the year 2000 (to increase generalisability, since levels of MVPA and/or ST might be different now than in the past, and because accelerometry became more widely used in research from the early 2000’s) up to March 2017. The literature search strategy used in MEDLINE is given in Table 1, and was adapted as required for the other four databases. Full literature search details are available from the corresponding author on request. The electronic search was complemented by reference tracking (forward and backward) of the eligible studies.

**Table 1:** Search strategy used for MEDLINE database.

1. exp Child/
2. exp Adolescent/
3. (child* or adolesc* or teen* or youth or girl* or boy*).tw.
4. (young adj1 (person or people)).tw.
5. 1 or 2 or 3 or 4
6. exp Exercise/
7. exp Motor Activity/
8. exp Sports/
9. exercis*.tw.
10. physical* activ*.tw.
11. (active adj2 (living or lifestyle)).tw.
12. sedentary behavi?r.tw.
13. exp Sedentary Lifestyle/
14. ((sedentary or sitting or screen or TV or television or computer or PC or video games) adj2 time).tw.
15. 6 or 7 or 8 or 9 or 10 or 11 or 12 or 13 or 14
16. exp Obesity/ or exp Overweight/
17. (overweight or obes*).tw.
18. exp Accelerometry/
19. exp Actigraphy/
20. acceleromet*.tw.
21. actigraph.tw.
22. activity monitor*.tw
23. (objective adj1 (measure* or monitor* or assess*)).tw.
24. 18 or 19 or 20 or 21 or 22 or 23
25. exp cardiovascular abnormalities/ or exp heart diseases/
26. exp Cardiovascular Abnormalities/
27. exp Heart Diseases/
28. "congenital heart disease".tw.
29. "Atrial Septal Defect".tw.
30. "Complete Atrioventricular Canal Defect ".tw.
31. "Ventricular Septal Defect".tw.
32. (Tetralogy adj2 Fallot).tw.
33. exp Asthma/
34. asthma.tw.
35. exp Respiratory Tract Diseases/
36. exp Respiratory Hypersensitivity/
37. exp Cystic Fibrosis/
38. (respiratory adj2 allerg*).tw.
39. "cystic fibrosis".tw.
40. wheez*.tw.
41. exp Bronchopulmonary Dysplasia/
42. lung diseases/ or exp alpha 1-antitrypsin deficiency/ or exp "cystic adenomatoid malformation of lung, congenital"/ or exp hepatopulmonary syndrome/ or exp hypertension, pulmonary/ or exp lung diseases, fungal/ or exp lung diseases, interstitial/ or exp lung diseases, obstructive/ or exp lung diseases, parasitic/ or exp lung injury/ or exp lung neoplasms/ or exp lung, hyperlucent/
43. "chronic lung disease".tw.
44. "chronic respiratory disease".tw.
45. exp diabetes mellitus, type 1/ or exp diabetes mellitus, type 2/
46. (diabetes adj1 mellitus).tw.
47. exp Leukemia/
48. Leukemia.tw.
49. exp Lymphoma/
50. Lymphoma.tw.
51. exp Neuroblastoma/
52. Neuroblastoma.tw.
53. "Wilms’ tumor".tw.
54. exp Central Nervous System Neoplasms/
55. exp Sleep Apnea Syndromes/
56. "sleep apnea".tw.
57. 16 or 17 or 25 or 26 or 27 or 28 or 29 or 30 or 31 or 32 or 33 or 34 or 35 or 36 or 37 or 38 or 39 or 40 or 41 or 42 or 43 or 44 or 45 or 46 or 47 or 48 or 49 or 50 or 51 or 52 or 53 or 54 or 55 or 56
58. 5 and 15 and 24 and 57
59. exp Adult/.
60. 58 not 59
61. limit 60 to (english language and yr = "2000 -Current")

#### Study selection

Titles, abstracts, and full-text articles were screened in duplicate for eligibility and disagreements were resolved through discussions with other reviewers when required. Reference lists of eligible studies were examined for potentially eligible studies. Reasons for exclusion are summarised in the study flow diagram and available in details from the corresponding author on request.

#### Data extraction and data synthesis

This review used a standard form for extracting relevant information from the eligible studies. The systematic review identified that the eligible studies fell logically into four categories: cardiovascular disease; respiratory disease; diabetes; malignancy. A fifth category (obesity) was identified but this is reported separately as noted above. Obesity was not included here because of the on-going debate about whether obesity is a disease or not. International recommendations for school-age children and adolescents specify at least 60 minutes of MVPA every day [5, 6], but in the eligible studies the achievement of MVPA recommendations was never operationalised in this way. In most studies, only the mean or median daily MVPA was provided (rather than achievement of MVPA recommendations on 7/7 days), and so this was used as a proxy for achievement of guideline recommendations in the present study.

Where suitable data for patients and healthy controls were reported, mean and standard deviation of MVPA and ST in minutes per day, and sample sizes for similar chronic disease conditions were combined in a random effects model accounting for heterogeneity between studies. Given the differing methods of determining MVPA levels obtained from accelerometers, differences in MVPA between patients and controls were generated as weighted standardised mean difference (SMD). While methodology (e.g. accelerometer model, accelerometry cut point and/or epoch) varied substantially between studies, within study comparisons are all based on the same methods. Separate meta-analyses were performed for MVPA and ST. Review Manager 5.2 was used for the quantitative analysis [21]. Some eligible studies recruited healthy control participants and measured MVPA and/or ST in the same way as in their patient group and at the same time (referred to here as studies with controls), while other studies compared patient data with other studies (e.g. published data) and are referred to here as studies with comparison groups; some studies simply reported patient data in relation to physical activity recommendations.

#### Quality assessment

Eligible articles were assessed for methodological quality using a 15-item quality assessment scale as shown in S2 Table, collapsed to 6 items for scoring, with higher scores suggesting higher study quality. Each eligible study was assessed independently by two authors (RE, JJR), and disagreements were resolved by discussion. The quality assessment scale was modified from the methodological quality assessment scale of Tooth *et al*. [22]. This is a reliable and valid 30-item tool for assessing the quality of observational studies, and was considered for use without modification initially. After careful reflection, modifications to the original scale were made to focus quality assessment on issues of particular importance to accelerometry measurement of PA. A modified Tooth tool has been used previously with several recent systemic reviews of PA studies with 8–17 items, that were usually collapsed to a smaller number of items for scoring [23–26].

## Results

### Identification of eligible studies

**Fig 1:**
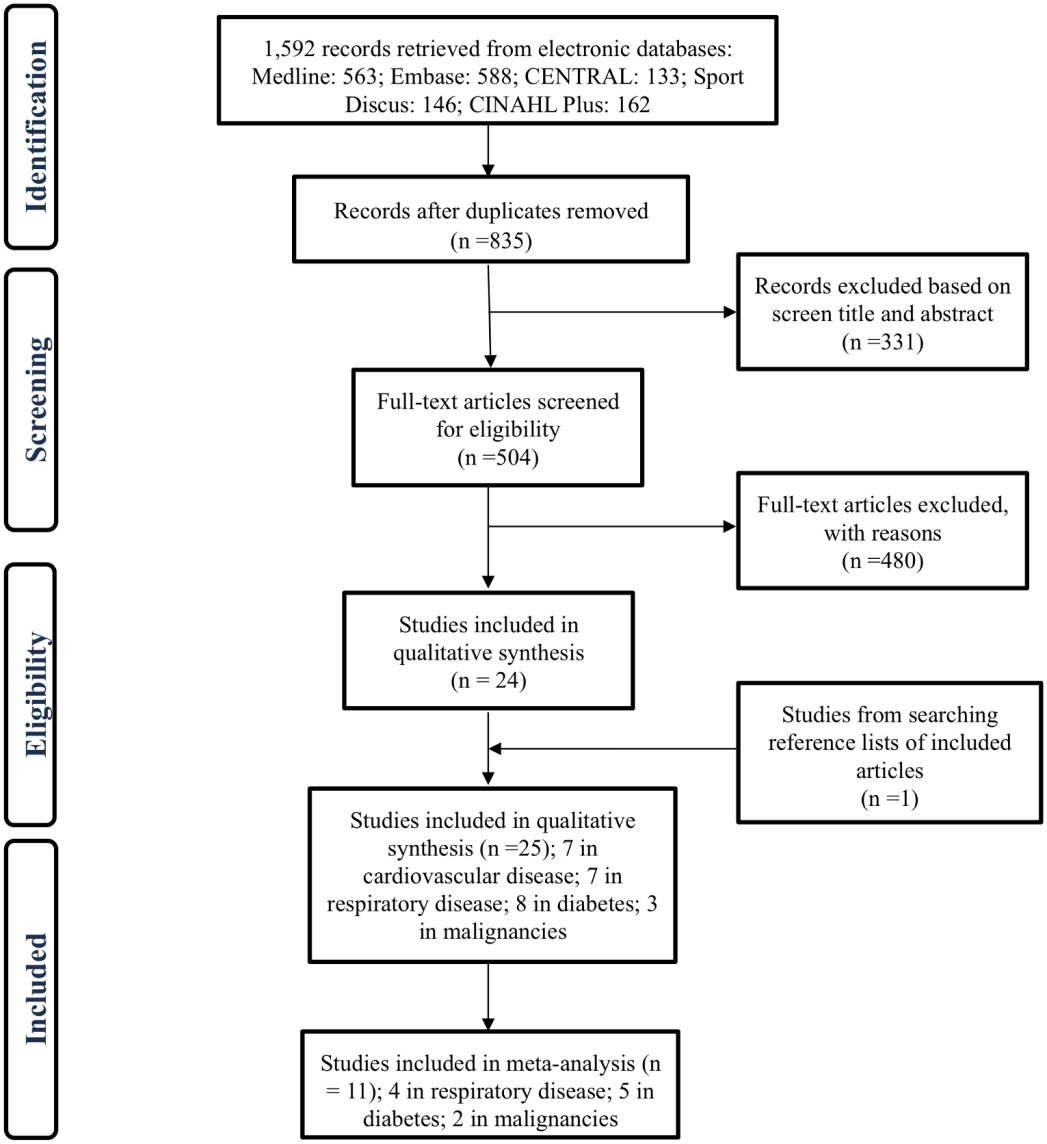
The PRISMA flow diagram with numbers of included and excluded articles at each step of the review process.

The PRISMA flow diagram with numbers of included and excluded articles at each step of the review process is provided in Fig 1. Tables 2–5 provide a brief summary of all studies included in this systematic review. Of the 1592 identified records from the five databases, 504 were selected for full text screening and of these, 24 met the inclusion criteria. Additionally, 1 study was identified and deemed eligible through searching references of eligible studies, bringing the final total to 25 eligible studies (7 in cardiovascular disease; 7 in respiratory disease; 8 in diabetes; 3 in malignancies) and 11 of these 25 studies were suitable for inclusion in meta-analysis (4 in those with respiratory disease; 5 in those with diabetes; 2 in those with malignancies). All eligible studies measured MVPA, and 16 out the 25 eligible studies compared levels of MVPA between patients with chronic disease and a healthy control group (referred to here as studies with controls), while the other 8 eligible studies compared data from patients with data from previously published studies of healthy children and adolescents (referred to here as studies with comparison groups); and two studies simply reported patient data in relation to recommendations; 14 of the 25 studies also provided data on ST, and 10 of these 14 studies compared ST in those with chronic disease with a healthy control group, while another 4 eligible studies compared data from patients with data from previously published studies of healthy children and adolescents comparison groups.

**Table 2:** Descriptive characteristics and levels of moderate-to-vigorous intensity physical activity and sedentary time in children with cardiovascular disease.

Reference*	Place and publication year	Sample group n, male% and age (yrs)	Control group n, male% and age (yrs)	Measurement	Accelerometry methods	MVPA	Sedentary time
**Banks et al** [27]	Canada, 2012	n: 20, (60% male)	N/A	Actigraph MTI, worn above the iliac crest for 4 days including 1 weekend day	Epoch = 60s	Median 8 (Q1 4—Q3 11) min/day	N/A
		Age: mean 11.8 (SD 3)			MVPA cut-points not clearly reported	MVPA lower in patients than healthy comparison groups	
						All patients failed to meet an average of 60 min/day MVPA	
**Banks et al** [28]	Canada, 2013	n: 50, (59% male)	N/A	Actical, worn above the iliac crest for 7 days	Epoch = 15s	Mean 52 (SD 20) min/day	N/A
		Age: range 6–12			MVPA ≥ 1600 cpm	MVPA lower in patients than healthy comparison groups	
						All patients failed to meet an average 60 min/day MVPA	
**Duncombe et al** [29]	Canada, 2016	n: 90, (54% male	N/A	Actigraph GT3X, worn on right hip for 7 days during waking hours	Epoch = 15s, Evenson cut-point [30]	Median 43 (IQ range 29–60) min/day	Median 70 (IQ range 61–76) % of waking time sedentary
		Age: mean 13.6 (SD 2.7)			MVPA ≥ 2296 cpm	MVPA similar in patients and healthy comparison groups	Sedentary time similar in patients and healthy comparison groups
					Sedentary time < 100 cpm	8% of patients achieved an average 60 min /day MVPA	
**Ewalt et al** [31]	USA, 2012	n: 21, (24% male)	n: 21, matched for age and gender	Actigraph 7164, worn on right hip for 7 days during waking hours	Epoch = 30s	Patients mean 71 (SD 50) min/day	Patients mean 399 (SD 107) min/day
		Age: mean 10.7 (SD 3.2)			MVPA cut-points not clearly reported	Control group mean 61 (SD 30) min/day	Control group mean 406 (SD 90) min/day
					Sedentary time ≤ 50 counts /30s	MVPA difference not significant (p = 0.2)	Sedentary time difference not significant (p = 0.7)
						33% of patients and 5% of control group achieved an average 60 min /day of MVPA	
**Gardner et al** [32]	Canada, 2016	n: 30, (46% male)	N/A	Actigraph GT3X, worn on right hip for 7 days during waking hours	Epoch = 15s, Evenson cut-point [30]	Median 40 (IQ range 27–57) min/day	Median 68 (IQ range 61–76) % of waking time sedentary
		Age: mean 10.7 (SD 3.2)			MVPA ≥ 2296 cpm	25% of patients achieved an average 60 min /day of MVPA	
					Sedentary time < 100 cpm		
Longmuir et al [33]	Canada, 2011	n: 63, (60% male)	N/A	Actical, worn above the iliac crest for 7 days during waking hours	Epoch = 15s	Mean 51 (19) min/day	N/A
		Age: range 5–11			MVPA ≥ 1,600 cpm	MVPA 4 to 5 times lower in patients than healthy comparison groups	
						3% of patients achieved an average of 60 min per day of MVPA	
**McCrindle et al** [34]	USA and Canada. 2007	n: 147, (62% male)	N/A	Actigraph MTI, worn for 4 days include 1 weekend day during waking hours	Epoch = 60s, Freedson cut-point [35]	Absolute MVPA not given	N/A
		Age: range 6–18				MVPA lower in patients than healthy comparison groups	
						38% of patients achieved an average of 60 min per day of MVPA	

cpm: counts per minutes; MVPA: moderate-to-vigorous physical activity; n: Number; N/A: No data included; S: Second; Data are expressed as mean (SD) unless otherwise; *Freedson MVPA cutpoint* [35] *calculated using the following equation*: *METS = 2*.*757 + (0*.*0015 x counts/min)–(0*.*08957 x age (yr))–(0*.*000038 x counts/min x age (yr))*

*One study [31] recruited healthy control and patient participants at the same time and measured MVPA and ST in the same way as (referred to here as studies with control group); 5 studies [27–29, 33, 34] compared patient data with other studies-previous published studies (referred to here as studies with comparison groups); and1 study [32] reported patient data in relation to recommendations.

**Table 3:** Descriptive characteristics and levels of moderate-to-vigorous intensity physical activity and sedentary time in children with chronic respiratory diseases.

Reference*	Place and publication year	Sample group n, male% and age (yrs)	Control group n, male% and age (yrs)	Measurement	Accelerometry methods	MVPA	Sedentary time
**Aznar et al** [36]	Spain, 2014	n: 47, (51% male)	**n**: 39; (59% male)	Actigraph GT3X, worn on right hip for 7 days during waking hours	Epoch = 15s, Evenson cut-point [30]	Patients mean 44 (SD 28) min/day	Patients mean 362 (SD 67) min/day
		Age: mean 12.0 (SD 3.0)	Age: mean 12.0 (SD 2.0)		MVPA ≥ 2296 cpm	Control group mean 54 (SD 15) min/day	Control group mean 484 (SD 85) min/day
					Sedentary time < 100 cpm	MVPA significantly lower in patient (p < 0.02)	Sedentary time was lower in patients (p < 0.001)
						2% of patients and 34% of control group achieved an average 60 min/ day of MVPA	
**Kilbride et al** [37]	Ireland, 2012	n: 16, (56% male)	n: 99, (48% male)	RT3 worn for 3 days	Epoch and MVPA cut-points not clearly reported	Patients mean boys 15 (SD 3); girls 16 (SD 3) min/day	N/A
		Age: range 10–12	Age: range 10–12		Sedentary time = 0–99cpm	Control group mean boys 23 (SD 6); girls 15 (SD 3) min/day	
						MVPA similar in patients and control group	
						All patients failed to meet an average of 60 minutes/day MVPA	
**Smith et al** [38]	Germany, 2016	n: 94, (56% male)	n: 590, (40% male)	Actigraph GT3X, worn on right hip for 7 days during waking hours	Epoch = 60s. Freedson cut-point [35]	Patients mean boys 41 (35) 11, 89; girls 43 (36) 14, 71 min/day	N/A
		Age: mean 15.6 (SD 0.5)	Age: mean 15.7 (SD 0.5)			Control group mean boys 46 (42) 19, 89; girls 38 (34) 13, 70 min/day	
**Tsai et al** [39]	USA, 2012	n: 27, (70% male)	n: 27, (59% male)	actigraph (Actiwatch 64 MM), worn non-dominant wrist for 7 days during waking hours	Epoch = 60s	Patients mean 265 (SD 83) min/day	Patients mean 87 (SD 48) min/day
		Age: range 9–11	Age: range 9–11		MVPA ≥ 700 cpm	Control group mean 308 (SD 97) min/day	Control group mean 77 (SD 27) min/day
					Sedentary time = 0–49 cpm	MVPA similar in patients and control group (p = 0.09)	Sedentary time difference not significant (p = 0.3)
						Patients and control group achieved an average of 60 min per day of MVPA	
**Vahlkvist et al** [40]	Denmark, 2010	n: 55	n: 154	RT3 worn for 4 weeks during 24 h a day	Epoch and cut-points not clearly reported	Patients mean of 32 (95% CI 5) min/day	Patients mean 1270 (95% CI 15) min/day
		Age: range 6–14	Age: range 6–14			Control group mean 34 (95% CI 3) min/day	Control group mean 1261 (95% CI 9) min/day
						MVPA similar in patients and control group	Sedentary time was similar in patient and control group
						Patients and control group failed to achieve an average of 60 min per day of MVPA	
**Van- Gent et al** [41]	Netherlands, 2007	n: 81, (58% male)	n: 202, (50% male)	Pam AM 100, worn on hip for 5 days during waking hours	Epoch = 60s	Patients mean 99 (95%, CI 80, 118) min/day	N/A
		Age: mean 9.4 (SD 0.8)	Age: mean 9.4 (SD 0.7)		MVPA cut-points not clearly reported	Control group mean 98 (95%, CI 85, 106) min/day	
						MVPA similar in patients and control group	
						Patients and control group achieved an average of 60 min per day of MVPA	
**Yiallouros et al** [42]	Cyprus, 2015	n: 36, (64% male)	n: 99, (60% male)	Actigraph worn on wrist for 7 days during waking hours	Epoch not clearly reported	Patients mean 15 (95% CI 10–21) min/day	Patients mean 939 (95% CI 915–963) min/day
		Age: range 8–9	Age: range 8–9		MVPA > 3200 cpm	Control group mean 16 (95% CI 14–19) min/day	Control group mean 927 (95% CI 915–938) min/day
					Sedentary time < 100 cpm	Similar in patients and control group	Similar in patients and control group

cpm: counts per minutes; MVPA: moderate-to-vigorous physical activity; n: Number; S: Second; Data are expressed as mean (SD) unless otherwise in Smith et al [38]: MVPA calculated as mean (Geometric mean) 5^th^, 95^th^ percentile, and Yiallouros et al [42]: MVPA Calculated as geometric means (95% CI) in min/day.; *Freedson MVPA cutpoint* [35] *calculated using the following equation*: *METS = 2*.*757 + (0*.*0015 x counts/min)–(0*.*08957 x age (yr))–(0*.*000038 x counts/min x age (yr))*

*Studies are recruited healthy control participants and measured MVPA and ST in the same way as in their patient participants and at the same time(referred to here as studies with control group).

**Table 4:** Descriptive characteristics and levels of moderate-to-vigorous intensity physical activity and sedentary time in children with diabetes mellitus.

Reference*	Place and publication year	Sample group n, male% and age (yrs)	Control group n, male% and age (yrs)	Measurement	Accelerometry methods	MVPA	Sedentary time
**Cuenca-Garcia et al** [43]	UK, 2012	n: 60, (67% male)	n: 37, (54% male)	Actigraph GT1M, worn for 7 days during waking hours	Epoch = 60s	Patients mean 28 (SD 21) min/day	N/A
		Age: mean 12.5 (SD 2.3)	Age: mean 12 (SD 2.5)		MVPA ≥ 3200 cpm	Control group mean 20 (SD 11) min/day	
						MVPA difference not significant (p = 0.06)	
**Kriska et al** [44]	USA, 2013	n: 669, (51.5% male)	N/A	Actigraph AM7164, worn on waist for 7 days during waking hours	Epoch = 60 s. Freedson cut-point [35]	10–14 year old mean boys 35 (SD 26); girls 27 (SD 18) min/ day	10–14 year old mean boys 495 (SD 144); girls 479 (SD 141) min/day
		Age: range 10–17			Sedentary time < 100 cpm	15–18 year old mean (boys 26 (SD 24); girls 8 (SD 9) min/day	15–18 year old mean boys 526 (SD 143); girls 546 (SD 143) min/day
						MVPA low in patients than healthy comparison groups	
						All patients failed to reach an average of 60 min/day MVPA	
**MacMillan et al** [45]	UK, 2014	n: 40, (50% male)	N/A	Actigraph GT3X, worn on waist for 7 days during waking hours	Epoch = 15s	Patients mean of 43 (SD 24) min/day	Patients mean 612 (SD 102) min/day
					MVPA ≥ 3200 cpm	MVPA similar in patients and healthy comparison groups	
		Age: mean 11.1 (SD 2.7)			Sedentary time < 100 cpm	5% of patients achieved an average of 60 min per day of MVPA	
**Maggio et al** [46]	Switzerland, 2010	n: 45	n: 85	Actigraph 6471, worn for 7 days during waking hours	Epoch not clearly reported	Patients mean 54 (SD 7) min/day	Patients mean 77% waking time sedentary
		Age: mean 10.7 (SD 0.4)	Age: mean 10.1 (SD 0.3)		MVPA > 2000 cpm	Control group mean 71 (SD 5) min/day	Control group mean 70% waking time sedentary
					Sedentary time < 500 cpm	The difference not significant (p = 0.07)	Significantly higher in patient (p < 0.01)
						39% of patient and 60% of control group achieved an average of 60 min/day of MVPA	
**Nguyen et al** [47]	Canada, 2015	n: 16; (n = 8) good glycemic control, (n = 8) poor glycemic control	n: 8	Actigraph GT1, worn on the right hip for 7 days during waking hours	Epoch = 3s, Evenson cut-point [30]	Patients with good glycemic control mean 46 (SD 16) min/day	N/A
		Age: range 8–16	Age: range 8–16		MVPA ≥ 2296 cpm	Patients with poor glycemic control mean 47 (SD 8) min/day	
					Sedentary time < 100 cpm	Control group mean 54 (SD 28) min/day	
						The difference not significant (p = 0.07)	
**Sarnblad et al** [48]	Sweden, 2005	n: 26, (100% female)	n: 49	Actigraph 6471 worn on the hip for 7 days during waking hours	Epoch = 60s	Patients mean of 56 (SD 20) min/da	Patients mean 443 (SD 60) min/day
		Age: range 12–19	Age: range 12–19		MVPA > 1952 cpm	Control group mean 60 (SD 23) min/day	Control group mean 390 (SD 27) min/day
					Sedentary time < 100 cpm	The difference not significant (p = 0.07)	Significantly higher in patient than control group (*P* = 0.002)
**Sundberg et al** [49]	Sweden, 2012	n:24, (50% male)	n: 26, (46.2% male)	Actiheart, for 7 days	Epoch = 60s	Absolute MVPA not given	Absolute sedentary time not given
		Age: mean boys 4.3 (SD 1.6); girls 4.7 (SD 1.9)	Age: mean boys 4.9 (SD 1.4); girls 4.4 (SD 1.8)		MVPA cut-point not clearly reported	MVPA significantly lower in patients than the control group (p = 0.02	Significantly higher in patient than control group (p = 0.03)
					Sedentary time < 100 cpm		
**Trigona et al** [50]	Switzerland, 2010	n: 32, (53% male)	n: 42, (40% male)	Actigraph 6471, worn on hip for at least 4 days during waking hours	Epoch = 60s	Patients mean 53 (95% CI 33–74) min/day	
		Age: range 6–17	Age: range 6–17		MVPA > 2000 cpm	Control group mean 77 (95% CI 58–97) min/day	
						Significantly lower in patients than the control group (p < 0.008)	
						35% of patients and 57% control group achieved an average of 60 min/day of MVPA	

cpm: counts per minutes; MVPA: moderate-to-vigorous physical activity; n: Number; S: Second; Data are expressed as mean (SD) unless otherwise; *Freedson MVPA cutpoint* [35] *calculated using the following equation*: *METS = 2*.*757 + (0*.*0015 x counts/min)–(0*.*08957 x age (yr))–(0*.*000038 x counts/min x age (yr))*.

*6 studies [43, 46–50] recruited healthy control participants and measured MVPA and ST in the same way as in their patient participants and at the same time (referred to here as studies with control group), and 2 studies [44, 45] compared patient data with other studies (referred to here as studies with comparison groups.

**Table 5:** Descriptive characteristics and levels of moderate-to-vigorous intensity physical activity and sedentary time in children with malignancies.

Reference*	Place and publication year	Sample group n, male% and age (yrs)	Control group n, male% and age (yrs)	Measurement	Accelerometry methods	MVPA	Sedentary time
**Aznar et al** [51]	Spain, 2006	n: 7, (57.1% male)	n: 7, (57.1% male	Actigraph MTI, worn on waist for 7 days during waking hours	Epoch = 60s, Freedson cut-point [35]	Patients mean 47 (SD 15) min/day	Patients mean 41 (SD 18) % of time sedentary
		Age: range 4–7	Age: range 4–7		Sedentary time ≤ 100 cpm	Control group mean 72 (SD 25) min/day	Control group mean 42 (SD 11) % of time sedentary
						Significantly lower in patient group than control group (p = 0.04)	Sedentary time was similar in patient and control group (p = 0.07)
						0% of patient, 57% control group achieved an average of 60 min/day of MVPA	
**Gotte et al** [52]	Germany, 2017	n: 28, (57% male)	N/A	Step Watch 3^™^ Monitor, worn for 7 days during waking hours	Epoch = 60s	Patients mean 4 (SD 5) min/day	N/A
		Age: range 11–15			MVPA cut-point not clearly reported	3% (n = 1) of patient achieved an average of 60 min/day of MVPA	
**Tan et al** [53]	Malaysia, 2012	n: 38	n: 38	Actical, worn on hip for 7 days during all the day	Epoch = 15s	Patients mean 20 (SD 28) min/day	Patients mean 1295 (SD 119) min/day
		Age: range 3–12	Age: range 3–12		MVPA cut off points not reported	Control group mean 168 (SD 56) min/day	Control group mean 925 (SD 111) min/day
					Sedentary time < 100 cpm	Significantly lower in patient group than control group (*p* < 0.01)	Significantly higher in patients than control group (*p* < 0.01)

cpm: counts per minutes; MVPA: moderate-to-vigorous physical activity; n: Number; S: Second; Data are expressed as mean (SD) unless otherwise; *Freedson MVPA cutpoint* [35] *calculated using the following equation*: *METS = 2*.*757 + (0*.*0015 x counts/min)–(0*.*08957 x age (yr))–(0*.*000038 x counts/min x age (yr))*

*2 studies [51, 53] recruited healthy control participants and measured MVPA and ST in the same way as in their patient participants and at the same time (referred to here as studies with control group), 1 study [52] reported patient data in relation to recommendations.

#### Study characteristics

Study samples: Eligible study sample sizes ranged from 14–699 with a total of 2062 participants with chronic disease and 1523 participants from healthy control groups. All studies were from high-income, developed nations. Measurement methods: A total of 17 out of the 25 eligible studies used the ActiGraph accelerometer to measure habitual MVPA and/or ST, though with a variety of different ActiGraph models and approaches to data collection and reduction. Of the remaining studies: three used the Actical [28, 33, 53]; two the RT3 “Triaxial Research Tracker” [37, 40]; one the PAM “Physical Activity Monitor” B.V. type AM 100 [41]; one the Actiheart (which combines accelerometry and heart rate monitoring) [49]; and one the Step Watch 3^™^ [52].

### MVPA in children and adolescents with chronic disease

The mean reported daily MVPA accumulated by children and adolescents with chronic disease across the 25 eligible studies ranged between 4 (SD 4) minutes/day [52] to 265 (SD 83) minutes/day [39].

**Children and adolescents with cardiovascular disease:** Seven of the 25 eligible studies (n = 442) examined MVPA in children and adolescents previously diagnosed with a congenital heart defect. This included children who had received different types of cardiac surgery [29, 31, 32], including complex surgery such as a fontan repair [28, 33, 34], or cardiac transplantation [27]. In all cases the patients were studied at least 6 months after surgery while well, clinically stable, free of acute illness, and living in the community. As summarised in Table 2, average daily MVPA in these studies ranged from a low of 8 (range, 4–11) min/day [27] to a high of 49 (range, 34–60) min/day [33]. In 6/7 of eligible studies reported mean daily time spent in MVPA in minutes and in six studies mean daily MVPA failed to reach the recommended 60 minutes. Only 1 out of 7 eligible studies included data from healthy control group, showing a higher MVPA level in the patient group compared to healthy control group although differences were not significant [31].

**Fig 2:**
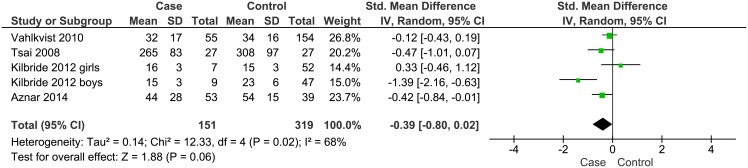
Forest plot of the comparison of moderate-to-vigorous intensity physical activity between children and adolescents with chronic respiratory diseases and healthy participants. SD: standard deviation; Std mean difference: Standardised mean difference; IV: Inverse variance; Random: random effect model; CI: 95% Confidence interval.

**Children and adolescents with chronic respiratory diseases:** Seven of the 25 eligible studies (n = 1013) investigated children and adolescents with chronic respiratory diseases; five studies in patients with asthma [38–42] and two in patients with cystic fibrosis [36, 37]. In all cases, the children were studied while clinically stable, free of acute illness, and while living in the community. The mean daily MVPA reported ranged from 15 (SD 3) min/day [37] to 265 (SD 8) min/day [39] as summarised in Table 3. In 2/7 eligible studies [39, 41] mean daily reported MVPA reached or exceeded the 60 minutes recommended, though this included one study with the exceptionally high reported levels of MVPA (30). Meta-analysis of all 4 studies indicated lower MVPA levels in the patient group compared to the healthy control group, approaching statistical significance. The standardised mean difference (SMD) was 0.39 (95% CI -0.80 to 0.02, p = 0.06). The heterogeneity was substantial with an I^2^ statistic of 68%, as shown in Fig 2.

**Fig 3:**
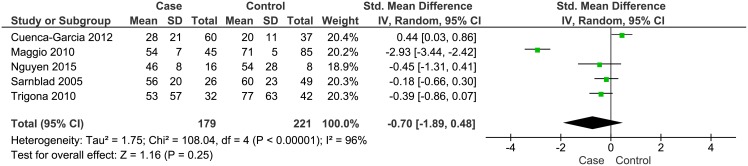
Forest plot of the comparison of daily moderate-to-vigorous intensity physical activity between children and adolescents with type 1 diabetes mellitus and healthy participants. SD: standard deviation; Std mean difference: Standardised mean difference; IV: Inverse variance; Random: random effect model; CI: 95% Confidence interval.

**Children and adolescents with diabetes:** Eight of the 25 eligible studies (n = 1323) involved children and adolescents with diabetes mellitus; 7 studies in children with type 1 diabetes [43, 45–50] and 1 in children with type 2 diabetes [44]. Again, in all cases the patients were studied while clinically stable and free of acute illness or diabetes complications, and while living in the community. As summarised in Table 4, the average daily MVPA reported for diabetic patients ranged from a low of 8 (SD 9) min/day [44] to a high of 56 (SD 20) min/day [48]. Of the 8 eligible studies, 7 reported mean daily MVPA in minutes and in all 7 of these studies MVPA was < 60 minutes. Patient MVPA was compared to healthy peers in 5 studies [43, 46–48, 50], all of which included patients with type 1 diabetes as shown in Fig 3. There was no evidence of a statistically significant difference in MVPA in patients compared to healthy controls (SMD -0.70, 95% CI -1.89 to 0.48, p = 0.25, n = 400). Case-control evidence on MVPA levels appears to be lacking for patients with type 2 diabetes.

**Fig 4:**
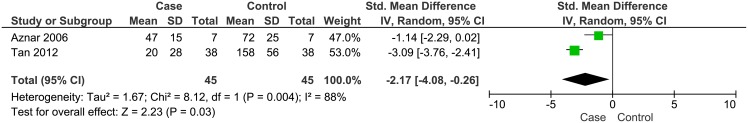
Forest plot of the comparison of daily moderate-to-vigorous intensity physical activity between children and adolescents with malignancies and healthy participants. SD: standard deviation; Std mean difference: Standardised mean difference; IV: Inverse variance; Random: random effect model; CI: 95% Confidence interval.

**Children and adolescents with malignancies:** Three studies, (n = 118) examined MVPA in those with malignancies including one study in acute lymphoblastic leukaemia on maintenance treatment [51], one in acute leukemia undergoing induction or consolidation chemotherapy [53] and a third in children and adolescents with different types of childhood malignancies [52]. In all of these studies, the participants had no other co-morbid conditions that would have been a contraindication for PA such as anemia, fever, or other difficulties with mobility. The mean daily MVPA achieved during these studies ranged from a low of 4 (SD 4) min/day [52] to a high of 47 (SD 15) min/day [51], as summarised in Table 5. In all three studies mean daily MVPA failed to reach the recommended 60 minutes. Two out the three studies included data from healthy participants and in both of these studies, the level of MVPA was significantly lower in children and adolescents with malignancies [51, 53]. Fig 4 shows the combined result, which suggests a standardised mean difference of 2.2 (95% CI -4.08 to -0.26, p = 0.03). Despite the apparent similarity between the studies the statistical heterogeneity was considerable with an I^2^ statistic of 88%. The heterogeneity noted could be due to differences in sample sizes, age and place of the studies, differences in the stage of treatment, or differences in accelerometer methodology.

### Sedentary time in children and adolescents with chronic disease

In this systematic review, 14 out of the 25 eligible studies reported on accelerometer measured ST, with a total of 1870 participants (those with chronic disease n = 1325; healthy control group n = 545). Of the 14 studies that measured ST, the chronic diseases studied were: cardiovascular disease—3 studies [29, 31, 32]; chronic respiratory diseases—4 studies [36, 39, 40, 42]; diabetes—5 studies [44–46, 48, 49]; malignancy—2 studies [51, 53].

As summarised in Tables 2–5, the mean daily time spent sedentary in these eligible studies ranged from a low of 87 (SD 48) min/day [39] to a high of 1295 (SD 119) min/day [53]. In 10 out of the 13 eligible studies, there was a healthy control group, and in 4/10 studies ST was significantly higher in those with chronic disease than in the healthy control groups [46, 48, 49, 53], in one study ST was significantly lower in the patient group compared to the healthy control group [36], and in 5 studies there was no significant group difference [31, 39, 40, 42, 51]. Suitable summary data for combining individual study data were only available for three studies (n = 355) in patients with chronic respiratory disease with findings indicating no statistically significant group difference in time spent sedentary (SMD -0.40, 95% CI -1.53 to 0.74, p = 0.49, I^2^ = 95%).

### Study quality assessment

Study quality assessment summaries are given in S3 Table: 3 studies scored 4/6; 13 scored 5/6; 9 scored 6/6 on study quality. Thus in general, studies were high methodological quality.

## Discussion

This systematic review provides evidence that children and adolescents with some chronic childhood diseases have lower than recommended levels of MVPA. In most of the eligible studies, daily MVPA averaged less than the 60 minutes/day recommended.

When comparing MVPA level between patients and healthy control or comparison groups, the findings indicated, within the limits of the available data, no marked differences for patients with type 1 diabetes, CVD and chronic respiratory diseases. In patients with leukemia compared to healthy control or comparison groups daily MVPA was significantly lower. With the respect to sedentary time the present review found that studies fairly consistently reported that children and adolescents with chronic disease accumulated a high amount of ST during their waking hours.

It should be noted that recommendations for MVPA state that 60 minutes per day is a minimum every day (e.g. usually operationalized as all 7 days in a week), but adherence to recommendations was not operationalized in this way in any of the 25 eligible studies. We therefore used a mean or median daily MVPA of 60 minutes as a proxy for compliance, though this is conservative because in many cases where 60 minutes/day was reached as an average, levels of MVPA would have fallen below 60 minutes/day on at least one of the monitored days.

Reasons for lower than recommended levels of MVPA are unclear. Children and adolescents with chronic disease may experience an over-protective care environment, a lack of supervised facilities/ opportunities for PA, and/or insufficient knowledge and self-efficacy about the types of PA suitable for the specific disease condition [54, 55]. Such socio-environmental influences could contribute to low daily MVPA and high ST. Healthcare professionals, parents/caregivers and schools may need to be provided with adequate information and training to be able to encourage and support children with chronic disease to engage in regular and appropriate MVPA. However, it should also be noted that reported levels of MVPA among healthy peers were also generally low in the eligible studies, so it may be that any constraints on PA which apply to healthy children and adolescents also apply equally to those with chronic disease.

We believe that the present study is the first systematic review to ask whether or not levels of MVPA are adequate in children and adolescents with chronic childhood disease. There are therefore no directly comparable studies, but we note that in healthy children, and particularly in healthy adolescents, there is concern that levels of MVPA are generally much lower than recommended. A global analysis by Hallal et al suggested that less than 20% of 13–15 year olds meet the recommendation of 60 minutes/ day of MVPA [56]. A recent pooling of international accelerometry data from nearly 21,000 healthy children and adolescents showed typically very low levels of adherence to the 60 minutes/day recommendation for MVPA [57], so it is perhaps not surprising that levels of MVPA among those with chronic disease were also found to be generally low in the present study.

We are also unaware of any previous systematic reviews of accelerometry measured ST among children and adolescents with chronic disease. Interpreting sedentary time data is even more problematic than interpreting the MVPA data in the present study because there are currently no evidence-based recommendations for accelerometer-measured ST.

Our review had a number of strengths. It was the first review to investigate objective levels of MVPA and ST in children and adolescents living with childhood chronic disease. Secondly, there are several methodological strengths to this study: in particular, studies were identified from an extensive search of the published literature conducted in a range of databases. The broad definition of search terms applied across multiple databases enabled the searching and identification across many potential studies. Restricting eligibility to accelerometry studies was important in increasing confidence in the objective measurement of MVPA [16, 58–60]. Finally, all included studies were in general rated as being of high or very high quality.

However, there are several weaknesses worth highlighting. Firstly, as studies had to be published in peer-reviewed journals in English, this may have excluded some relevant evidence. Studies included in our review investigated MVPA and ST in children and adolescents with chronic childhood disease. However, we excluded some other common medical conditions where significant alterations in activity might have been expected because of the nature of the condition e.g. musculo-skeletal and neurological disease, and we also excluded studies of patients with acute illness or injury requiring or associated with confinement or bed-rest. Future reviews should consider these other groups, and also consider the PA and ST of children and adolescents with the many chronic diseases not included in the present review. Our initial scoping review found that objectively measured PA data were available for only a few chronic disease groups and so the present review focused on those.

All eligible studies were from high-income developed nations. We therefore lack data from low-middle income countries where the prevalence of many childhood chronic disease will be common and lack of resources may limit medical care [61]. Most of the included studies were based on relatively small samples of children with chronic disease (n 14–699) and their power to estimate habitual MVPA, or to distinguish between MVPA of patients and comparison group participants, might have been limited, and their representativeness was rarely clear. Our method for assessing the quality of eligible studies has been used in variously adapted forms in a number of other recent accelerometry systematic reviews [23–26], and used 15 items, but the process of collapsing these 15 items to a six-item scale might have reduced the possibility of identifying differences in quality between studies. Eligible studies made comparisons with healthy peers in a wide variety of ways (control groups; comparison groups; reference to recommendations). Use of control groups was considered ideal, but restricting our synthesis to only those studies would have reduced a small evidence base to an even smaller evidence base, so this was not done.

Further, the eligible studies varied substantially in terms of the accelerometers used, and even where the same accelerometer was used the methods varied in a number of potentially important accelerometer data reduction decisions e.g. the definition of a monitoring epoch [43, 59]; the number of hours and days of data constituting a valid data set [44, 46]; MVPA and ST accelerometer cut-points; and criteria for the inclusion or exclusion of non-wear time [36, 45]. These differences between studies are likely to have produced meaningful differences in MVPA and ST estimates [13] and they make it difficult to compare across studies. For the present review, the level of heterogeneity between eligible studies was high when combining data in meta-analysis across studies. However, in the case of all eligible studies the methods used for patient and control/comparison groups were identical, so comparison *within-studies* remain meaningful.

An example of how the choice of accelerometer cut-point could affect conclusions reached by individual studies is that studies using lower accelerometer cut-points to define MVPA tended to report higher levels of habitual MVPA than those which used lower accelerometer cut-points to define MVPA. Tsai et al [39], for example used an Actigraph accelerometer cut-off of ≥ 700 counts per minute to define MVPA in children with asthma. This cut-off point is well below the cut-points used more commonly and which are more evidence based (based on calibration studies such as the Evenson et al cut-point of 2296 counts per minute [30]; or the Puyau et al cut-point of 3200 counts per minute [62]). The very low accelerometer cut-point used by Tsai et al almost certainly led to the very high estimate of 265 (SD 83) minutes of daily MVPA [39], and could lead to the erroneous conclusion that levels of MVPA among children with asthma are extremely high.

## Conclusions

In summary, this systematic review found that overall (habitual) MVPA levels are well below international recommendations in at least some groups of children and adolescents with chronic childhood diseases. The present review suggests that management of pediatric chronic conditions should place greater emphasis on MVPA, and patients with at least some chronic diseases are probably not currently benefiting from the health and non-health benefits that MVPA can bring. Time spent sedentary is often higher than in the comparison groups, and probably too high in many patients, but this is difficult to interpret in the absence of health-related recommendations for accelerometer measured ST in children and adolescents. This valuable information about the MVPA and ST levels in children with chronic disease may help to stimulate improving PA guidelines, and improving PA for these children. The need for more extensive research in this area, including intervention studies of the impact of increased MVPA levels on health related outcomes, is clear.

## Supporting information

S1 File PRISMA 2009 checklist preferred reporting items for systematic reviews and meta-analyses. (DOC)

S2 File Systematic review protocol. (DOCX)

S1 Table Inclusion and exclusion criteria for selection of studies. MVPA: Moderate-to-Vigorous Intensity Physical Activity; PA: physical activity; ST: sedentary time.
(DOCX)

S2 Table Study quality assessment criteria, modified from Tooth *et al* [22]. MVPA: Moderate-to-Vigorous Intensity Physical Activity.
(DOCX)

S3 Table Methodological quality assessment of the included studies. + Indicates that a criterion was satisfied; − indicates that a criterion was not satisfied. 1, described of Sample recruitment?; 2, description of the sample.?; 3, Attrition of sample?; 4, Data collection and reduction?; 5, MVPA definition given?; 6, MVPA Results given?; * Studies are listed based on diseases groups.
(DOCX)
