# Supplementary material for: Accelerometer measured levels of moderate-to-vigorous intensity physical activity and sedentary time in children and adolescents with chronic disease: A systematic review and meta-analysis
Source: PLoS One. 2017 Jun 22;12(6):e0179429. doi: 10.1371/journal.pone.0179429 (PMC5480890; doi:10.1371/journal.pone.0179429)
Supplement: S2 File — (DOCX) [file pone.0179429.s002.docx]

**Title:**

**Accelerometer measurement of physical activity and / or sedentary behaviours of children and adolescents with chronic diseases: Systematic review and meta-analysis**

# ****Review question(s):****

Are accelerometer measurement of physical activity (PA) and/ or sedentary behaviours (SB) of children and adolescents with chronic childhood diseases similar to children who do not have a chronic disease?

Based on accelerometer measurement of physical activity (PA) are children/ adolescents with chronic childhood diseases meeting current recommendations for physical activity (PA)?

# Methods

## Search strategy

The search strategy focuses on four key elements: children and/or adolescents, PA and/or SB, measured objectively by accelerometer, and common chronic diseases (obesity, chronic cardiovascular diseases, chronic respiratory diseases, diabetes mellitus and cancer).

We will gather our materials for systematic literature review from the following electronic databases: Medline, Cochrane library, EMBASE, SPORT Discus and CINAHL for articles published in peer reviewed journals. In addition, we will identify articles by hand searching the references lists of published reviews and related cited articles than include all studies that will find. Only full text articles in English language will be included in this review. Because accelerometers started to be used in physical activity and sedentary behaviour research over the last 15 years, the literature search will be restricted to articles published between 2000 until 2015. No limitations will be placed on publication sample size and country of origin. Reasons for exclusion of studies will be recorded.

### Inclusion criteria:

All studies will have to meet all of the following criteria as per the PICOS principles to be eligible for inclusion in this systematic review.

#### Population:

Participants from any gender, had to be aged 18 years old or younger to include both children and adolescents living with common chronic childhood disease.

Chronic diseases are defined as conditions lasting at least 6 months, which were diagnosed by a doctor or generally accepted objective criteria for that particular condition and included in the article under examination. We will restrict the clinical populations included to five common childhood chronic conditions:

i. Obesity: studies will be included where the obese paediatric population was defined as having a body mass index (BMI) ≥ 95th percentile for children of the same sex and age, or the child or adolescent BMI conceptually equivalent to or 30 kg/m^2^ International Obesity Task Force (IOTF),or >+2SD according to British 1990 growth references (UK90) or other national or international BMI reference data, e.g. from the Centers for Disease Control (CDC) charts and World Health Organization (WHO) charts for ages.

Studies which included only the overweight group, and which combine overweight and obese groups will be excluded.

**i**i. Chronic cardiovascular diseases: studies to be included will be those in children/adolescents with:

Congenital Heart Diseases (CHD) most common types, both cyanotic and acyanotic (Atrial Septal Defect (ASD), Co-arctation of the Aorta (CoA), Complete Atrioventricular Canal defect (CAVC), Ventricular Septal Defect (VSD), Tetralogy of Fallot (TOF), Transposition of the Great Arteries (TGA).

Studies of children with hypertension (High Blood Pressure) and cardiac muscle disease (e.g. cardiomyopathies) will also be included.

iii. Chronic Respiratory diseases: studies to be included will be those in children/adolescents with:

Asthma, viral induced wheeze, sleep apnea syndrome, pulmonary hypertension, cystic fibrosis, broncho-pulmonary dysplasia (BPD), chronic lung disease (CLD) that diagnosed by doctor or generally accepted objective criteria.

iv. Diabetes Mellitus: studies to be included will be those in children/adolescents with:

Type1 Diabetes mellitus (T1DM) or Type 2 Diabetes mellitus (T2DM) that meet WHO diagnostic criteria for diabetes; fasting plasma glucose ≥ 7.0mmol/l (126mg/dl) or 2–h plasma glucose ≥ 11.1mmol/l (200mg/dl).

v. Cancer: studies to be included will be those in children/adolescents with blood and solid tumours (Leukemia, Lymphoma, Neuroblastoma, Wilms’ tumors and CNS tumors).

#### Intervention or exposure:

**Overall (habitual) physical activity (MVPA-expand-)** and/ SB **measured by accelerometer for at least** 6 hours or more per day over three consecutive days or more.

#### Comparison:

Where applicable, levels of physical activity, particularly moderate and vigorous physical activity (MVPA) and or sedentary behaviour will be compared between children with chronic disease vs healthy controls or with recommendations in current guidelines.

#### Outcomes:

1. Primary outcomes:

The main outcome is accelerometer measured overall (habitual) MVPA.

1. Secondary outcomes:

A secondary outcome is accelerometer measured overall (habitual) total volume of PA, .e.g. accelerometer counts per minute or per 15 seconds. A final secondary outcome is total habitual sedentary time as measured by accelerometer.

#### Study design:

Including cross-sectional, longitudinal (retrospective, prospective), and case-control studies. Intervention studies will be eligible for inclusion if baseline data can be extracted.

#### Type of publication:

Peer-reviewed full-text articles published in English in academic journals

#### Period of research:

The literature search will be restricted to articles published from 2000 until current year (2015).

Studies will not be restricted according to study sample size or country of origin.

### Exclusion criteria:

The present review will exclude all studies that examined participants were aged 19 years or above or participants ≤18 years with acute medical diseases or condition that may have impacted their physical activity levels. We will also exclude studies where participants had any limitation to walking for example, orthopaedic injury, or were using wheelchair for mobility.

Studies that used subjective methods of PA measurement (child report, parent, or carer proxy report) and studies that used objective and direct observation methods apart from accelerometry (e.g. heart rate monitors, pedometers) will be excluded.

We also will exclude all studies that measured habitual physical activity (MVPA) for less than 6 hours per day and studies that collected PA data over two days or less. Further, we will exclude all studies that focused only on specific periods of the day (e.g. school activity only or outdoor activity only or weekend or weekday activity only).

## Study Selection:

Titles and abstracts and full-text articles will be screened in duplicate for eligibility. Potentially eligible studies will be cross-checked between reviewers and disagreements will be resolved through discussions. Where the two reviewers do not agree, a third reviewer will abbreviate.

## Data extraction:

Reviewers will use an electronic data extraction form for extracting relevant information from the studies. Extracts will be compared in meetings and discrepancies resolved through discussion. Then eligible papers that meet inclusion criteria will be assessed again by two authors and evidence tables compiled reporting on author name, year of publication, country studied, aim of study, recruitment procedure, study participants (sample size, age range), study design, setting of intervention, accelerometer protocol (type and model of accelerometer, requested number of days the accelerometers were to be worn, and outcome), other health measurements, key findings and results.

## Quality assessment:

The risk of bias will be independently assessed by two review authors and cross-checked and discussed to resolve disagreement where required. We will use a tool that was published by the Critical Appraisal Skills Programme United Kingdom ([www.casp-uk.net](http://www.casp-uk.net)) to assess the quality of included studies.

## Data synthesis:

Studies that are not suitable to be included in a meta-analysis will be described narratively. Studies reporting similar outcome measures (e.g. physical activity or sedentary behaviour) in a similar population (e.g., obese children vs healthy control children) on a similar scale (e.g., minutes/day) will be combined in a meta-analysis. Separate analysis will be conducted for continuous and binary data (e.g., meeting MVPA guideline vs not meeting the guidelines).

Where the data allow, we will calculate mean differences and standard deviations of PA and SB levels comparing a) chronically ill children with health children and b) PA levels of chronically ill children against the current PA recommendations.

### Subgroup Analysis:

Subgroup analyses are principally intended to investigate sources of heterogeneity within a meta-analysis in relation to factors which potentially impact on outcomes. Our data will be divided into five main groups according to the diseases that investigated (obesity, chronic cardiovascular diseases, chronic respiratory disease, diabetes mellitus and cancer). If the number of included studies allows further sub group analysis () will focus on:

- Gender: boys vs. girls
- Age group: preschool children age group vs. school age group

## Sensitivity analysis:

We will investigate the influence of study characteristics on the robustness of the review results by conducting sensitivity analyses. We will remove trials from the analysis and perform a re-analysis with the remaining studies when studies were of low methodological quality.
