# Supplementary material for: Accelerometer measured levels of moderate-to-vigorous intensity physical activity and sedentary time in children and adolescents with chronic disease: A systematic review and meta-analysis
Source: PLoS One. 2017 Jun 22;12(6):e0179429. doi: 10.1371/journal.pone.0179429 (PMC5480890; doi:10.1371/journal.pone.0179429)
Supplement: S1 Table — MVPA: Moderate-to-Vigorous Intensity Physical Activity; PA: physical activity; ST: sedentary time. (DOCX) [file pone.0179429.s003.docx]

S1 Table: Inclusion and exclusion criteria for selection of studies

| **Inclusion criteria** | **Exclusions criteria** |
| --- | --- |
| Participants aged ≤ 19 years | Participants aged >19. |
| Children and adolescent with common chronic diseases: cardiovascular diseases, chronic respiratory diseases diabetes and malignancy. Chronic diseases defined as medical conditions lasting at least 3 months, diagnosed by a doctor or by using generally accepted objective criteria. | Children and adolescents with acute diseases or conditions (e.g. post-surgery) that may have impacted their PA levels.  Participants who had any limitation to their PA e.g. orthopaedic injury. |
| **MVPA** and/ ST **measured by** accelerometer **methods for at least** 6 h/day for three consecutive days or more. | - Studies that used subjective methods of MVPA **and ST** measurement (child report, parent, or carer proxy report).  - Studies that used objective and direct observation methods apart from accelerometers (e.g. heart rate monitors, pedometers).  - Studies which measured MVPA **or ST** for less than 6 hours per day.  - Studies that collected PA data over two days or less.  - Studies that focused only on specific periods of the day (e.g. school activity only or outdoor activity only). |
| Articles published in English from 2000.  Published in peer-reviewed journals. | Articles published before 2000.  Review papers without original data, studies using previously reported data. |
| Human studies | Animal studies. |

**MVPA: Moderate-to-Vigorous Intensity Physical Activity; PA: physical activity; ST: sedentary time.**
