# Supplementary material for: Accelerometer measured levels of moderate-to-vigorous intensity physical activity and sedentary time in children and adolescents with chronic disease: A systematic review and meta-analysis
Source: PLoS One. 2017 Jun 22;12(6):e0179429. doi: 10.1371/journal.pone.0179429 (PMC5480890; doi:10.1371/journal.pone.0179429)
Supplement: S2 Table — MVPA: Moderate-to-Vigorous Intensity Physical Activity. (DOCX) [file pone.0179429.s004.docx]

S2 Table: Study Quality Assessment Criteria, modified from Tooth *et al.* ([22](#_ENREF_22)).

| Criterion | Definition | Mark Allocation |
| --- | --- | --- |
| Sample recruitment. | Sample: How were they recruited.  Time: What time of year was the study conducted.  Place: Where did the recruitment take place. | 1 point for listing 3 criteria. |
| Sample description of the sample (number, age, gender). | Number of participants recruited  Mean age of participants.  Percentage male and female. | 1 point for listing all 3 criteria. |
| Attrition | Number of participants recruited and the number actually measured. | 1 point for listing both criteria. |
| Data collection and reduction. | Type of device; length of epoch; no of days of active commuting specified as minimum; duration of monitoring time; monitor placement; data reduction decisions. | 1 point for listing 3 criteria. |
| MVPA definition given. | Methods of MVPA definition e.g. accelerometer cut-points or other method given. | 1 point for listing 1 criteria. |
| Results. | Adequate description of numbers actually analysed, with summary MVPA data. | 1 point for listing both criteria. |

**MVPA: Moderate-to-Vigorous Intensity Physical Activity.**
