# Supplementary material for: Accelerometer measured levels of moderate-to-vigorous intensity physical activity and sedentary time in children and adolescents with chronic disease: A systematic review and meta-analysis
Source: PLoS One. 2017 Jun 22;12(6):e0179429. doi: 10.1371/journal.pone.0179429 (PMC5480890; doi:10.1371/journal.pone.0179429)
Supplement: S3 Table — + Indicates that a criterion was satisfied; − indicates that a criterion was not satisfied. 1, described of Sample recruitment?; 2, description of the sample.?; 3, Attrition of sample?; 4, Data collection and reduction?; 5, MVPA definition given?; 6, MVPA Results given?; * Studies are listed based on diseases groups. (DOCX) [file pone.0179429.s005.docx]

S3 Table: Methodological quality assessment of the included studies.

|  | Quality Assessment Criteria, Items 1-6 | | | | | | | |
| --- | --- | --- | --- | --- | --- | --- | --- | --- |
| *Reference | | 1 | 2 | 3 | 4 | 5 | 6 | Total score |
| Banks, 2012 | | - | + | + | + | + | + | 5/6 |
| Banks, 2013 | | - | + | + | + | + | + | 5/6 |
| Duncombe, 2016 | | + | + | + | + | + | + | 6/6 |
| Ewalt, 2012 | | + | + | + | + | + | + | 6/6 |
| Gardner, 2016 | | - | + | + | + | + | + | 5/6 |
| Longmuir, 2011 | | - | + | + | + | + | + | 5/6 |
| McCrindle, 2007 | | - | + | + | + | + | + | 5/6 |
| Aznar, 2014 | | - | + | + | + | + | + | 5/6 |
| Kilbride, 2012 | | - | + | + | + | + | + | 5/6 |
| Smith, 2016 | | + | + | + | + | + | + | 6/6 |
| Tsai, 2012 | | + | + | + | + | + | + | 6/6 |
| Vahlkvist, 2012 | | - | - | + | + | + | + | 4/6 |
| Van Gent, 2007 | | + | + | + | + | + | + | 6/6 |
| Yiallouros, 2015 | | + | - | + | + | + | + | 5/6 |
| Cuenca-Garcia | | - | + | + | + | + | + | 5/6 |
| Kriska, 2013 | | + | + | + | + | + | + | 6/6 |
| MacMillan, 2014 | | + | + | + | + | + | + | 6/6 |
| Maggio, 2010 | | - | - | + | + | + | + | 4/6 |
| Nguyen, 2015 | | - | + | + | + | + | + | 5/6 |
| Sa ̈rnblad, 2005 | | + | + | + | + | + | + | 6/6 |
| Sundberg, 2012 | | + | + | + | + | + | + | 6/6 |
| Trigona, 2010 | | - | + | + | + | + | + | 5/6 |
| Aznar, 2006 | | + | + | + | + | + | + | 6/6 |
| Gotte 2015 | | - | + | + | + | + | + | 5/6 |
| Tan, 2012 | | + | - | + | + | + | + | 5/6 |

+ Indicates that a criterion was satisfied; − indicates that a criterion was not satisfied. 1, described of Sample recruitment?; 2, description of the sample.?; 3, Attrition of sample?; 4, Data collection and reduction?; 5, MVPA definition given?; 6, MVPA Results given?; * Studies are listed based on diseases groups.
